# Genetic variation in *XRCC1*, sun exposure, and risk of skin cancer

**DOI:** 10.1038/sj.bjc.6602174

**Published:** 2004-09-21

**Authors:** J Han, S E Hankinson, G A Colditz, D J Hunter

**Affiliations:** 1 Channing Laboratory, Department of Medicine, Brigham and Women's Hospital, and Harvard Medical School, 181 Longwood Ave., Boston, MA 02115, USA; 2 Program in Molecular and Genetic Epidemiology, Harvard School of Public Health, 665 Huntington Ave., Boston, MA 02115, USA; 3 Department of Epidemiology, Harvard School of Public Health, 665 Huntington Ave., Boston, MA 02115, USA

**Keywords:** XRCC1, sunlight, skin cancer

## Abstract

The *XRCC1* gene is involved in the base excision repair pathway. We assessed the associations of polymorphisms and haplotypes in *XRCC1* with skin cancer risk in a nested case–control study within the Nurses' Health Study (219 melanoma, 286 squamous cell carcinoma (SCC) and 300 basal cell carcinoma (BCC), and 873 controls). We genotyped four haplotype-tagging single-nucleotide polymorphisms (Arg194Trp, C26602T, Arg399Gln, and Gln632Gln). There was no significant difference in frequency distribution between cases and controls for any of the five inferred common haplotypes. We observed that the 399Gln allele was inversely associated with SCC risk. This inverse association was only seen among those who had five or more lifetime sunburns, those with a family history of skin cancer, and those in the highest tertile of cumulative sun exposure in a bathing suit, but not among those with low risk defined by these risk factors. We also observed a significant association of the carriage of 194Trp allele with increased SCC risk, which was modified by family history of skin cancer. These two polymorphisms were not associated with BCC or melanoma risk. Our data suggest that the Arg194Trp and Arg399Gln polymorphisms may be differently associated with skin cancer risk according to exposure dose and skin cancer type.

The genotoxic effect of sunlight exposure has been demonstrated in the aetiology of both melanoma and nonmelanocytic skin cancer (Armstrong *et al*, 1997; English *et al*, 1997; Ravanat *et al*, 2001). Ultraviolet (UV) radiation is capable of causing a wide range of lesions in DNA. In addition to DNA photoproducts generated upon the direct absorption of UVB (280–320 nm), UVA (320–400 nm) can indirectly cause oxidative DNA damage in the form of oxidation of guanine (8-hydroxyguanine) and single-strand breaks via reactive oxygen species generated after the absorption of light energy by cellular chromophores (Hall and Johnson, 1996; Brash, 1997; Kielbassa *et al*, 1997). UVB can also induce oxidative DNA damage to a lesser extent (Kvam and Tyrrell, 1997).

Base excision repair (BER) is responsible for repair of oxidative DNA damage and single-strand breaks (Memisoglu and Samson, 2000; Nilsen and Krokan, 2001). It has been shown that modulation of BER alters cellular sensitivity to UVA but not to UVB (Kim *et al*, 2002). The XRCC1 protein is involved in the BER pathway. Although XRCC1 has no known enzymatic activity, there are three distinct domains that are sites of interaction with DNA polymerase *β* (Kubota *et al*, 1996; Marintchev *et al*, 2000), poly(adenosine diphosphate (ADP)-ribose) polymerase (PARP), and DNA ligase III (Callebaut and Mornon, 1997; Zhang *et al*, 1998; Thompson and West, 2000). This suggests that XRCC1 may act as a nucleating factor in BER by bringing different DNA repair components together at the site of action. A number of single-nucleotide polymorphisms (SNPs) in *XRCC1* have been identified (Shen *et al*, 1998; Mohrenweiser *et al*, 2002). These polymorphisms may alter the BER capacity, and in turn confer genetic predisposition to skin cancer. We assessed whether candidate polymorphisms and haplotypes in the *XRCC1* gene are associated with skin cancer risk (melanoma, basal cell carcinoma (BCC), and squamous cell carcinoma (SCC)) in a nested case–control study within the Nurses' Health Study. We further investigated the gene–environment interactions between *XRCC1* genetic variation and sun exposure-related risk factors on skin cancer risk.

## MATERIALS AND METHODS

### Study population

The Nurses' Health Study was established in 1976, when 121 700 females registered nurses between the ages of 30 and 55, residing in 11 larger US states, completed a self-administered questionnaire on their medical histories and baseline health-related exposures. Updated information has been obtained by questionnaires every 2 years. Between 1989 and 1990, blood samples were collected from 32 826 volunteers of the cohort members. Eligible cases in this study consisted of women with incident skin cancer from the subcohort who gave a blood specimen, including SCC and BCC cases with a diagnosis anytime after blood collection up to June 1, 1998 and melanoma cases (including *in situ* cases) up to June 1, 2000 with no previously diagnosed skin cancer. All available pathologically confirmed melanoma and SCC cases and 300 self-reported BCC cases randomly selected from about 2600 available self-reported BCC cases were included. The validity of self-report of BCC is high in this medically sophisticated population (90%) (Colditz *et al*, 1986; Hunter *et al*, 1990). All the SCC and BCC cases had no history of melanoma diagnosis. A common control series (case : control=1 : 1) was randomly selected from participants who gave a blood sample and were free of diagnosed skin cancer up to and including the questionnaire cycle in which the case was diagnosed. One control was matched to each case by year of birth (±1 year) and race (Caucasian, Asian, Hispanic, others). At the time we selected cases and controls, 47 cases and 69 controls were deceased. In order to obtain additional information by supplementary questionnaires, we randomly selected a second matched living control when the first control was deceased, and collected supplementary questionnaires from these second living controls. The nested case–control study consisted of 219 melanoma cases (including 77 *in situ* cases), 286 SCC cases, 300 BCC cases, and 874 matched controls. Owing to the absence of African-American cases, one African-American control was excluded to avoid potential population stratification. We mailed to 758 living cases and 804 living controls a supplementary questionnaire on lifetime sun exposure and other skin cancer risk factors. In all, 695 cases responded, 15 cases refused to participate, and 48 cases did not respond after three mailings (participation rate=92%). Among controls, 713 responded, nine refused, and 82 did not respond (participation rate=89%). The study protocol was approved by the Committee on Use of Human Subjects of the Brigham and Women's Hospital, Boston, MA, USA.

### Exposure data

Information regarding skin cancer risk factors was obtained from the prospective biennial questionnaires and the retrospective supplementary questionnaire. Information on natural hair color and childhood and adolescent tendency to sunburn or tan was asked in the 1982 prospective questionnaire; ethnic group in the 1992 questionnaire. The retrospective supplementary questionnaire consisted of questions in three major areas: (1) pigmentation, constitutional, and susceptibility factors; (2) history of residence (states and towns), sun exposure habits, and severe sunburns at different ages; and (3) family history of skin cancer (father, mother, and siblings). In addition, the 11 states of residence of cohort members at baseline were grouped into three regions: Northeast (CT, MA, MD, NJ, NY, and PA), Northcentral (MI and OH), and West and South (CA, TX, and FL). The reliability of the measurements obtained on both the prospective and retrospective data (natural hair color, childhood tendency to tan, and burn) was approximately in the same magnitude in terms of correlation coefficient and mean change among the cases and controls and the odds ratios (ORs) based on both questionnaires were similar, indicating that the retrospective assessment was not likely to substantially bias the estimates of risk in this study, at least for these variables.

In order to estimate sunlight exposure for each subject, a UV database for 50 US states was developed. The database used reports from the Climatic Atlas of the US, which reported mean daily solar radiation (in Langleys) at the earth's surface for weather stations around the country. The records of average annual solar radiation for January and July were extracted to represent winter and summer radiation, respectively. The mean solar radiation for each state was derived from the average of UV values measured in weather stations within that state, and both summer and winter radiation indices were developed for each state. A cumulative lifetime sun exposure was developed by combining the UV database and the information obtained from the supplementary questionnaires. Questions about sun exposure while wearing a bathing suit were used to define a cumulative lifetime sun exposure variable for this behavior.

### SNP identification

As described previously (Han *et al*, 2003), we selected haplotype-tagging SNPs for the *XRCC1* gene, using data derived from the resequencing of the exons and adjacent intronic and noncoding regions of the gene in a multiple-ethnicity group of 90 samples from the NIH DNA Polymorphism Discovery Resource available from the Coriell Institute for Medical Research (Collins *et al*, 1998; Mohrenweiser *et al*, 2002). Four haplotype tagging, SNPs were selected for the five common haplotypes inferred from 17 common SNPs (>1% allele frequency) of the gene. We genotyped these four SNPs (C26304T(Arg194Trp), C26602T, G28152A(Arg399Gln), and G36189A(Gln632Gln)) in the present case–control study of mostly Caucasian women.

### Laboratory assays

Genotyping was performed by the 5′ nuclease assay (TaqMan®), using the ABI PRISM 7900HT Sequence Detection System (Applied Biosystems, Foster City, CA, USA), in 384-well format. TaqMan® primers and probes were designed using the Primer Express® Oligo Design software v2.0 (ABI PRISM). Laboratory personnel were blinded to case–control status and blinded quality control samples were inserted to validate genotyping procedures; concordance for the blinded samples was 100%. Primers, probes, and conditions for genotyping assays are available upon request.

### Statistical analysis

We performed haplotype estimation using the Partition-Ligation Expectation Maximization Algorithm (Qin *et al*, 2002). We used a *χ*^2^ test to assess whether the *XRCC1* genotypes were in Hardy–Weinberg equilibrium and to determine *P*-values for differences in haplotype frequencies between cases of each type and common controls. We used a common control series in data analysis to increase the statistical power. Unconditional logistic regression was employed to calculate OR and 95% confidence interval (CI) to assess the risks of the three types of skin cancer for *XRCC1* genotypes. A test for trend was calculated across the three genotypes for each polymorphism. The results did not change substantially in the matched analysis for each of the three types of skin cancer with their own matched controls. We used a likelihood ratio test (LRT) to evaluate heterogeneity in the effects of the *XRCC1* genotypes on different types of skin cancer in polytomous logistic regression models (Marshall and Chisholm, 1985). To summarise multiple variables, we constructed a multivariate confounder score to create a constitutional susceptibility index for skin cancer (Miettinen, 1976). Briefly, we applied the logistic regression coefficients from a multivariate model including age, race, natural skin colour, natural hair colour, child or adolescent tendency to burn, and the number of palpably raised moles on arms, to each individual's values for the latter four of these variables, and summed the values to compute a susceptibility risk score in the logit scale. We used this score to define women with low, intermediate, and high constitutional susceptibility based on tertiles among controls. In the gene–environment interaction analyses, the number of severe lifetime sunburns and cumulative sun exposure with a bathing suit were also categorised into tertiles with cutpoints based on the distribution of controls.

To test the statistical significance of interactions between the environmental exposures and the *XRCC1* genotype, we compared the models that included terms for all combinations of the *XRCC1* genotype and levels of environmental exposure to the models with indicator variables for the main effects only (nominal LRT). We also modelled *XRCC1* genotypes as ordinal variables and environmental exposures as continuous variables to assess the statistical significance of interactions by testing the significance of a single multiplicative interaction term (ordinal LRT). All *P*-values were two-sided.

## RESULTS

### Descriptive characteristics of cases and controls

The mean ages at diagnosis of melanoma, SCC, and BCC cases were 63.4, 64.7, and 64.0 years, respectively, and that of controls, 64.5 years. Self-reported major ethnicity was similar between cases and controls (cases *vs* controls: Caucasian, 85.3 *vs* 85.7%; others, 11.7 *vs* 11.6%; one Asian melanoma case and one control; one Hispanic SCC case and two controls). Significantly positive associations were observed of lighter natural skin color, lighter natural hair colour, child or adolescent tendency to burn, and the number of palpably raised moles on arms with the risk of all three types of skin cancer. The risk for the highest tertile of the susceptibility score was about three-fold higher for SCC and BCC and four-fold higher for melanoma, compared to the lowest tertile. A family history of skin cancer was a risk factor for the three types of skin cancer. Cases of each skin cancer type were more likely to have used sunlamps or attended tanning salons. The number of lifetime severe sunburns that blistered was significantly associated with all three types of skin cancer. Women in the West and South regions were more likely to be diagnosed with SCC or BCC compared to those in Northeast.

### *XRCC1* and skin cancer risk

The genotype distributions of the four haplotype-tagging SNPs were in Hardy–Weinberg equilibrium among controls. The five common haplotypes inferred from these four SNPs accounted for 99% of the alleles of the present study population of mostly Caucasian women (Table 1

**Table 1:** Frequencies of PLEM inferred haplotypes in cases and controls

**Haplotypes**				**Frequency**						
**C26304T (Arg194Trp)**	**C26602T**	**G28152A (Arg399Gln)**	**G36189A (Gln632Gln)**	**Melanoma cases (*n*, %)**	***P*-value**	**SCC cases (*n*, %)**	***P*-value**	**BCC cases (*n*, %)**	***P*-value**	**Common controls (*n*, %)**
0	0	0	0	47 (11.8)	0.50	73 (13.8)	0.06	51 (9.8)	0.59	168 (10.7)
1	0	0	0	26 (6.5)	0.78	40 (7.5)	0.30	33 (6.3)	0.87	97 (6.2)
0	1	0	0	14 (3.5)	0.40	16 (3.0)	0.12	25 (4.8)	0.71	69 (4.4)
0	0	1	0	147 (36.9)	0.54	169 (31.7)	0.14	187 (35.9)	0.77	555 (35.3)
0	0	0	1	163 (40.9)	0.42	233 (43.8)	0.79	223 (42.9)	0.91	680 (43.2)

Haplotype frequencies from our observed genotypes were estimated using PLEM. Five common haplotypes were listed. The *P*-values for differences in haplotype frequencies between cases and controls were determined by the two-sample proportion test incorporating standard error from PLEM output. ‘0’ stands for common allele; ‘1’ stands for rare allele. Three additional rare haplotypes were also inferred (all less than 0.5% in cases or controls).

). Three rare haplotypes were also estimated. There was no significant difference in frequency distribution in cases and controls for any haplotype. The four haplotype-tagging SNPs were mutually exclusive to each other on these five common haplotypes. In other words, individuals with the variant allele of one polymorphism carried the haplotype that only harbored this variant, but not others. Therefore, the carriage of a polymorphism can be viewed as the carriage of the corresponding haplotype in the evaluation of main effect of polymorphic sites and gene–environment interactions.

We therefore evaluated the four haplotype-tagging SNPs in relation to skin cancer risk (Table 2

**Table 2:** *XRCC1* genotypes and skin cancer risk

		**Melanoma**			**SCC**			**BCC**		
**Genotype**	**Controls**	**Cases**	**Multivariate OR^b^**	**Multivariate OR^c^**	**Cases**	**Multivariate OR^b^**	**Multivariate OR^c^**	**Cases**	**Multivariate OR^b^**	**Multivariate OR^c^**
Arg194Trp^d^										
wt/wt	764	185	1.00	1.00	240	1.00	1.00	260	1.00	1.00
het	93	30	1.24 (0.80–1.93)	1.28 (0.79–2.06)	44	1.44 (0.98–2.11)	1.51 (1.01–2.28)	34	1.09 (0.73–1.63)	1.26 (0.82–1.93)
var/var	6	0			0			3		
Heterogeneity^e^	0.56									
										
C26602T^d^										
wt/wt	770	198	1.00	1.00	261	1.00	1.00	261	1.00	1.00
het	68	15	0.83 (0.46–1.48)	1.01 (0.55–1.88)	17	0.68 (0.39–1.18)	0.73 (0.41–1.30)	28	1.14 (0.72–1.80)	1.12 (0.68–1.83)
var/var	4	0			0			0		
Heterogeneity^e^	0.25									
										
Arg399Gln										
wt/wt	345	76	1.00	1.00	128	1.00	1.00	108	1.00	1.00
het	351	99	1.26 (0.90–1.77)	1.17 (0.81–1.67)	112	0.85 (0.63–1.14)	0.84 (0.62–1.15)	124	1.13 (0.84–1.52)	1.08 (0.79–1.48)
var/var	119	29	1.10 (0.68–1.77)	0.83 (0.49–1.39)	33	0.74 (0.48–1.14)	0.61 (0.39–0.97)	35	0.94 (0.61–1.45)	0.84 (0.53–1.33)
Trend			0.42	0.77		0.13	0.04		0.93	0.68
Heterogeneity^e^	0.18									
										
Gln632Gln										
wt/wt	277	72	1.00	1.00	89	1.00	1.00	98	1.00	1.00
het	406	103	0.97 (0.69–1.36)	1.06 (0.74–1.52)	142	1.10 (0.81–1.50)	1.19 (0.86–1.65)	147	1.02 (0.76–1.38)	1.06 (0.78–1.46)
var/var	170	38	0.87 (0.56–1.36)	0.97 (0.61–1.54)	53	0.98 (0.66–1.45)	1.00 (0.66–1.50)	52	0.87 (0.59–1.28)	0.87 (0.58–1.31)
Trend			0.57	0.95		0.98	0.86		0.54	0.61
Heterogeneity^e^	0.82									

a The number of participants does not sum to total women because of missing data on genotype.

b Unconditional logistic regression adjusted for the matching variables: age and race (Caucasian, non-Caucasian).

c Unconditional logistic regression adjusted for the matching variables, constitutional susceptibility score (tertiles), family history of skin cancer (yes/no), the number of lifetime severe sunburns which blistered (none, 1–5, 6–11, >11), sunlamp use or tanning salon attendance (yes/no), cumulative sun exposure while wearing a bathing suit (tertiles), and geographic region.

d The OR was calculated to compare heterozygote and homozygous variant combined with wildtype.

e Likelihood ratio test to evaluate heterogeneity in the effects of the *XRCC1* genotypes on different types of skin cancer in polytomous logistic regression models adjusted for variables in the multivariate model C.

). We observed a significantly increased risk of SCC among 194Trp carriers. As compared with noncarriers, the multivariate OR was 1.51 for women with at least one 194Trp allele (95% CI 1.01–2.28). We observed that the 399Gln allele was associated with significantly decreased risk of SCC. Compared with women with 399 Arg/Arg genotype, women with Arg/Gln and Gln/Gln genotypes had multivariate ORs of 0.84 (95% CI 0.62–1.15) and 0.61 (95% CI 0.39–0.97) for the risk of SCC, respectively (*P* for trend, 0.04). Our data showed that the two variants 194Trp and 399Gln were not in the same common haplotype. Carriage of either variant allele was not associated with altered skin cancer risk compared with being homozygous wild type for both alleles (data not shown). No overall associations between C26602T and Gln632Gln and three types of skin cancer risks were found. There was no significant heterogeneity in the main effect of each genotype on the three types of skin cancer (Table 2). It is noteworthy that there is another nonsynonymous polymorphism Arg280His, which is in 100% genotype concordance with C26602T among the 90 individuals from the NIH DNA Polymorphism Discovery Resource resequenced by the NIEHS Environmental Genome Project (http://egp.gs.washington.edu).

### *XRCC1* Arg399Gln, risk factors, and skin cancer risk

We evaluated potential interactions between the Arg399Gln polymorphism and lifetime severe sunburns, family history of skin cancer, cumulative sun exposure with a bathing suit, and geographic region on SCC risk (Table 3

**Table 3:** Interaction between *XRCC1* Arg399Gln and risk factors on SCC risk

	**Arg/Arg**		**Arg/Gln**		**Gln/Gln**		
	**Cases/controls**	**OR**	**Cases/controls**	**OR**	**Cases/controls**	**OR**	***P*, trend**
Lifetime severe sunburns^b^							
Never	17/110	1.00	20/89	1.56 (0.75–3.28)	8/27	2.06 (0.77–5.56)	0.12
1–4	39/87	2.75 (1.42–5.35)	26/94	1.64 (0.81–3.31)	11/28	2.18 (0.88–5.38)	0.38
⩾5	47/72	3.19 (1.63–6.22)	41/85	2.36 (1.21–4.61)	11/36	1.18 (0.48–2.89)	0.04
	*P* for interaction, 0.05 for nominal test; 0.008 for ordinal test						
							
Family history of skin cancer^c^							
No	80/269	1.00	70/257	0.90 (0.61–1.32)	25/83	0.96 (0.56–1.63)	0.71
Yes	48/76	1.75 (1.09–2.81)	42/94	1.27 (0.78–2.04)	8/36	0.40 (0.17–0.93)	0.001
	*P* for interaction, 0.02 for nominal test; 0.01 for ordinal test						
							
Cumulative sun exposure with a bathing suit^d^							
Low	25/94	1.00	22/87	0.83 (0.42–1.64)	9/31	0.96 (0.40–2.35)	0.74
Intermediate	29/89	1.09 (0.57–2.05)	33/96	1.27 (0.68–2.37)	7/25	0.99 (0.37–2.65)	0.66
High	54/89	2.58 (1.43–4.67)	41/88	1.74 (0.94–3.22)	14/40	1.10 (0.50–2.45)	0.02
	*P* for interaction, 0.48 for nominal test; 0.17 for ordinal test						
							
Geographic Region^e^							
Northeast	50/145	1.00	47/160	0.93 (0.58–1.51)	17/52	0.91 (0.46–1.78)	0.96
Northcentral	19/68	0.96 (0.51–1.81)	13/48	0.84 (0.41–1.74)	4/18	0.56 (0.17–1.84)	0.19
West and South	34/56	2.11 (1.19–3.72)	27/60	1.32 (0.73–2.40)	9/21	1.07 (0.44–2.62)	0.12
*P* for interaction, 0.81 for nominal test; 0.26 for ordinal test							

a The number of participants does not sum to total women because of missing data on genotype.

b Unconditional logistic regression adjusted for the matching variables, constitutional susceptibility score (tertiles), family history of skin cancer (yes/no), sunlamp use or tanning salon attendance (yes/no), cumulative sun exposure with a bathing suit (tertiles), and geographic regions.

c Unconditional logistic regression adjusted for the matching variables, constitutional susceptibility score (tertiles), the number of lifetime severe sunburns which blistered (none, 1–5, 6–11, >11), sunlamp use or tanning salon attendance (yes/no), cumulative sun exposure with a bathing suit (tertiles), and geographic regions.

d Unconditional logistic regression adjusted for the matching variables, constitutional susceptibility score (tertiles), family history of skin cancer (yes/no), the number of lifetime severe sunburns which blistered (none, 1–5, 6–11, >11), sunlamp use or tanning salon attendance (yes/no), and geographic regions.

e Unconditional logistic regression adjusted for the matching variables, constitutional susceptibility score (tertiles), family history of skin cancer (yes/no), the number of lifetime severe sunburns which blistered (none, 1–5, 6–11, >11), sunlamp use or tanning salon attendance (yes/no), and cumulative sun exposure with a bathing suit (tertiles).

). A significantly positive association of the number of lifetime severe sunburns with SCC was seen among women with the 399Arg/Arg genotype (five or more *vs* never, OR 3.19; 95% CI 1.63–6.22), and this excess risk was attenuated among those who carried the Gln allele. The Gln allele was significantly inversely associated with SCC risk among those who had five or more severe sunburns in their lifetime (*P* for trend, 0.04), but positively associated with SCC risk among women who never had severe sunburns (*P* for trend, 0.12). This interaction was statistically significant (*P* for ordinal test, 0.008; *P* for nominal test, 0.05). The 399Gln allele was not significantly associated with lifetime sunburns among controls. The interaction pattern described above was limited to those in the West and South regions, but not among those in Northeast and Northcentral regions (data not shown).

We observed that the inverse association of *XRCC1* 399Gln on SCC risk was significantly stronger among the individuals with a family history of skin cancer (*P* for trend, 0.001) than women without family history of skin cancer (*P* for trend, 0.71; *P* for ordinal interaction, 0.01; *P* for nominal interaction, 0.02) (Table 3). It was noteworthy that the 399Gln allele was associated with family history of skin cancer (*P*, *χ*^2^ for trend, 0.05) among controls.

In addition, a significantly inverse association of the 399Gln allele on SCC risk was only seen among women in the highest tertile of exposure (*P* for trend, 0.02), whereas no apparent association between the 399Gln allele and SCC risk was observed among women in the lowest or intermediate tertile (Table 3). The interaction was not statistically significant. There was no significant interaction between geographic region of residence and the Arg399Gln polymorphism on SCC risk, even though the inverse association of the Arg399Gln polymorphism and SCC risk was more apparent in the Northcentral region and West and South regions.

We also observed a significant interaction between the Arg194Trp polymorphism and family history of skin cancer on SCC risk (*P* for interaction, 0.05). Compared with 194Trp noncarriers without family history, the 194Trp carriers with a family history of skin cancer had a multivariate OR of 3.45 (95% CI, 1.64–7.25), whereas the multivariate OR was 1.16 (95% CI, 0.71–1.92) for 194Trp carriers without family history, and 1.19 (95% CI, 0.85–1.67) for 194Trp noncarriers with family history. No interactions were observed between the Arg194Trp and other risk factors on SCC risk. We did not observe any notable interactions between the two polymorphisms Arg399Gln and Arg194Trp and the above risk factors on BCC or melanoma risk.

## DISCUSSION

In this nested case–control study, we observed a significantly inverse association of the *XRCC1* 399Gln allele with SCC risk, along with the finding that the association of the 399Gln allele with SCC risk was significantly modified by a family history of skin cancer and the number of lifetime severe sunburns. We also observed a significant association of the carriage of *XRCC1* 194Trp allele with increased SCC risk, which was modified by a family history of skin cancer. The nested case–control design, high follow-up rate, and high response rate for the retrospective supplementary questionnaire strengthen the validity of this study.

The Arg399Gln polymorphism has recently drawn considerable attention because of its location in the region of the BRCT-I interaction domain of XRCC1 with PARP. Associations have been reported between the 399Gln allele and higher DNA adduct levels (Lunn *et al*, 1999; Duell *et al*, 2000; Matullo *et al*, 2001) and higher sister chromatid exchange frequency (Duell *et al*, 2000). However, in an *in vitro* transfection experiment, the wild-type and variant alleles equally complemented both the single-strand break repair defect and the sensitivity to methyl methanesulphonate in *XRCC1*-deficient EM9 cells, suggesting that the 399Gln variant retained a substantial level of function (Taylor *et al*, 2002). A positive association of the 399Gln allele with the risk of SCC was observed among women who never had severe blistering sunburns. Given the above data suggesting the reduced repair activity of the 399Gln allele, our data showed that the positive association of the 399Gln variant and SCC risk occurred in the context of low levels of DNA damage. In contrast, among women with five or more severe sunburns, a significantly inverse association was observed between the 399Gln allele and the risk of SCC. A possible explanation is that, when challenged by an overwhelmingly high dose of exposure, keratinocytes with impaired DNA repair capacity may accumulate excessive damage, thus inducing apoptosis and decreasing the risk of SCC. The lifetime sunburn variable combines exposure intensity and biological response to sun exposure. We also observed a significantly inverse association of the 399Gln allele with SCC risk among women with higher risk according to family history of skin cancer or cumulative sun exposure while wearing a bathing suit, along with a relatively null association among women with lower risk.

We did not observe significant effect modification of the *XRCC1* Arg399Gln on the relation of these risk factors with melanoma risk. UVA has been reported to have low capacity to induce melanoma compared to UVB in opossum models (Robinson *et al*, 1998, 2000), which suggests that UVA-induced oxidative DNA damage repaired by BER pathway may be less important than UVB-induced photoproducts in melanoma development in mammals. In contrast to keratinocytes, which are eliminated by apoptosis when severely damaged by UV radiation, melanocytes have low levels of cell cycling and proliferation and a limited capacity to undergo apoptosis, perhaps due to a high content of antiapoptotic proteins in melanocytes (Danno and Horio, 1987; Gilchrest *et al*, 1999; Bowen *et al*, 2003). Therefore, compared to melanocytes, keratinocytes have lower tolerance level of DNA damage and lower apoptotic threshold, which make the apoptosis pathway a protective mechanism for SCC, especially when cells are challenged by excess amount of DNA damage. The less-differentiated basal cells, that give rise to BCC, also have less susceptibility to apoptosis than keratinocytes (Gilchrest *et al*, 1999).

We evaluated the association of the Arg399Gln polymorphism and SCC risk according to geographic regions in our study. The overall inverse association of the 399Gln allele on SCC risk was more evident among individuals in Northcentral, West and South regions. An overall significant interaction between sunburns and the Arg399Gln genotype was only apparent among women in West and South region, with a significantly inverse association among the women with three or more sunburns. This interaction was not seen among women in North regions, which indicated that the overall interaction was driven by the individuals in the high-sun exposure region. These data suggest that the apoptosis due to the 399Gln allele is more apparent among individuals with relatively high sun exposure.

One previously published study assessed the Arg399Gln polymorphism and nonmelanoma skin cancer risk in a population-based case–control study in New Hampshire (Nelson *et al*, 2002). Compatible with our results, these authors found that the *XRCC1* 399 Gln/Gln genotype was related to reduced risks of both BCC (OR 0.7; 95% CI 0.4–1.0) and SCC (OR 0.6; 95% CI 0.3–0.9). However, these authors observed an inverse association of the 399Gln allele among women with two or less sunburns, but not among those with three or more sunburns. We did not observe this interaction pattern either in the whole study or among women in the Northeast region.

We observed that the *XRCC1* 194Trp allele was significantly associated with increased risk of SCC. Given the consistent relation of the 194Trp allele with the reduced cancer risk of bladder, lung, breast, and stomach observed in some case–control studies (Goode *et al*, 2002), the 194Trp allele is presumably associated with an enhanced DNA repair capacity. For keratinocytes, where the apoptosis pathway may serve as a major protective mechanism against UV-induced DNA damage, an enhanced DNA repair capacity may help cells escape apoptosis but, consequently, leave excess DNA damage unrepaired in the genome, potentially leading to subsequent mutation and increased cancer risk. No association has been found between Arg194Trp and altered levels of biomarkers of DNA damage (Lunn *et al*, 1999). We had limited power to detect the interactions between this variant and risk factors on the three types of skin cancer due to the low frequency of this allele.

Our data suggest that the association of the *XRCC1* Arg399Gln polymorphism with skin cancer risk may vary according to exposure dose and cancer type. We observed some evidence that the 399Gln allele was related to a decreased risk of SCC, but not that of melanoma or BCC. The effect modification of the 399Gln allele on SCC risk associated with sunburn suggests that oxidative DNA damage by the UVA predominantly present in sunlight may play an important role in the development of SCC. Given the number of comparisons, the findings should be interpreted with caution and confirmed by other studies.
